# Correction: Guo et al. Bioinformatic Analysis of the Value of Mitophagy and Immune Responses in Corneal Endothelial Dysfunction. *Curr. Issues Mol. Biol.* 2025, *47*, 670

**DOI:** 10.3390/cimb48020181

**Published:** 2026-02-05

**Authors:** Ruilin Guo, Chenjia Xu, Yi Yu, Minglu Ma, Xiaojuan Dong, Jing Wu, Chen Ouyang, Jie Ling, Ting Huang

**Affiliations:** 1 State Key Laboratory of Ophthalmology, Zhongshan Ophthalmic Center, Sun Yat-sen University, Guangdong Provincial Key Laboratory of Ophthalmology and Visual Science, Guangzhou 510060, China; linlin128@126.com (R.G.); yuyi57@mail2.sysu.edu.cn (Y.Y.); mingluma@126.com (M.M.); qingerdong@126.com (X.D.); wujing@gzzoc.com (J.W.); oyc1991@163.com (C.O.); lingjie2018@163.com (J.L.); 2 Ningbo Eye Institute, Ningbo Eye Hospital, Wenzhou Medical University, Ningbo 315040, China; xuchj1189@163.com

## Text Correction

There was an error in the original publication [1]. In Section 2.5 (PPI Network Analysis), the original text mistakenly referred to three algorithms by incorrect terms. The sentences and abbreviations should be corrected.

Original (incorrect):

“Hub genes (top 10 scores) were mined from this network via five topological algorithms: Matthews correlation coefficient (MCC) [23], differential metabolic network construction (DMNC), maximal neighborhood coefficient (MNC), edge percolated component (EPC), and Degree [24,25].”

A correction has been made to Section 2.5: PPI Network Analysis:

“Hub genes (top 10 scores) were mined from this network via five topological algorithms: Maximal Clique Centrality (MCC) [23], Density of Maximum Neighborhood Component (DMNC), Maximum Neighborhood Component (MNC), Edge Percolated Component (EPC), and Degree [24,25].”

## Abbreviations

Original (incorrect):

MCC	Matthews correlation coefficient
DMNC	Differential metabolic network construction
MNC	Maximal neighborhood coefficient

A correction has been made to the Abbreviations:

MCC	Maximal Clique Centrality
DMNC	Density of Maximum Neighborhood Component
MNC	Maximum Neighborhood Component

The authors state that the scientific conclusions are unaffected. This correction was approved by the Academic Editor. The original publication has also been updated.
